# Characterizing patient compliance over six months in remote digital trials of Parkinson’s and Huntington disease

**DOI:** 10.1186/s12911-018-0714-7

**Published:** 2018-12-20

**Authors:** Shani Cohen, Zeev Waks, Jordan J. Elm, Mark Forrest Gordon, Igor D. Grachev, Leehee Navon-Perry, Shai Fine, Iris Grossman, Spyros Papapetropoulos, Juha-Matti Savola

**Affiliations:** 1 Advanced Analytics Department, Intel, 94 Em Hamoshavot Road, Petah Tikva, Israel; 2 Department of Public Health Sciences, Medical University of South Carolina, 135 Cannon St., Suite 303, PO Box 250835, Charleston, SC 29425 USA; 3 Teva Branded Pharmaceutical Products R&D, Inc, 41 Moores Rd., Frazer, Petah Tikva, PA 19355 USA; 4 Guide Pharmaceutical Consulting, LLC, Millstone Township, NJ 08535 USA; 5 Teva Pharmaceutical Industries Ltd, 12 Hatrufa St, 4250483 Netanya, Israel; 6 Data Science Institute, Interdisciplinary Center, 1 Kanfei Nesharim St, 4610101 Herzliya, Israel; 7 CAMP4 Therapeutics, One Kendall Square, Bldg 1400 West, 3rd Floor, Cambridge, MA 02139 USA; 8 Massachusetts General Hospital, Department of Neurology, Boston, USA; 9 Teva Pharmaceuticals International GmbH, Elisabethenstrasse 15, 4051 Basel, Switzerland

**Keywords:** Remote clinical trials, Digital trials, Compliance, Wearables, Sensors, Smartphones, Parkinson’s disease, Huntington disease

## Abstract

**Background:**

A growing number of clinical trials use various sensors and smartphone applications to collect data outside of the clinic or hospital, raising the question to what extent patients comply with the unique requirements of remote study protocols. Compliance is particularly important in conditions where patients are motorically and cognitively impaired. Here, we sought to understand patient compliance in digital trials of two such pathologies, Parkinson’s disease (PD) and Huntington disease (HD).

**Methods:**

Patient compliance was assessed in two remote, six-month clinical trials of PD (*n* = 51, Clinician Input Study funded by the Michael J. Fox Foundation for Parkinson’s Research) and HD (*n* = 17, sponsored by Teva Pharmaceuticals). We monitored four compliance metrics specific to remote studies: smartphone app-based medication reporting, app-based symptoms reporting, the duration of smartwatch data streaming except while charging, and the performance of structured motor tasks at home.

**Results:**

While compliance over time differed between the PD and HD studies, both studies maintained high compliance levels for their entire six month duration. None (− 1%) to a 30% reduction in compliance rate was registered for HD patients, and a reduction of 34 to 53% was registered for the PD study. Both studies exhibited marked changes in compliance rates during the initial days of enrollment. Interestingly, daily smartwatch data streaming patterns were similar, peaking around noon, dropping sharply in the late evening hours around 8 pm, and having a mean of 8.6 daily streaming hours for the PD study and 10.5 h for the HD study. Individual patients tended to have either high or low compliance across all compliance metrics as measured by pairwise correlation. Encouragingly, predefined schedules and app-based reminders fulfilled their intended effect on the timing of medication intake reporting and performance of structured motor tasks at home.

**Conclusions:**

Our findings suggest that maintaining compliance over long durations is feasible, promote the use of predefined app-based reminders, and highlight the importance of patient selection as highly compliant patients typically have a higher adherence rate across the different aspects of the protocol. Overall, these data can serve as a reference point for the design of upcoming remote digital studies.

**Trial registration:**

Trials described in this study include a sub-study of the Open PRIDE-HD Huntington’s disease study (TV7820-CNS-20016), which was registered on July 7th, 2015, sponsored by Teva Pharmaceuticals Ltd., and registered on Clinicaltrials.gov as NCT02494778 and EudraCT as 2015–000904-24.

**Electronic supplementary material:**

The online version of this article (10.1186/s12911-018-0714-7) contains supplementary material, which is available to authorized users.

## Background

The widespread global use of smartphones and connected sensor devices, in parallel with their continuously increasing capabilities, has begun to impact the clinical trial ecosystem. Digital wearable and non-wearable devices containing electronic sensors such as accelerometers, gyroscopes, and photosensors can now track diverse biomarkers including heart rate, blood pressure, heart rate variability, lung function, and gait to various degrees of accuracy [1]. Complementarily, smartphone applications enable frequent and facilitated interaction with patients in the form of reminders, self-reporting of drug intake, or acquisition of electronic patient-reported outcomes [2].

Remote data collection via connected devices may provide added value to clinical trials [3]. Statistically, remote technologies increase data collection frequency, consequently providing insight on variability, requiring less extrapolation, and potentially increasing study power. These insights typically rely on signal processing and machine learning methods developed using sensor collected (big) data. Additionally, sensors, while not exclusively used in remote settings, are inherently objective compared to clinician scoring of disease status [4]. Home-based monitoring may also be more objective from the patient perspective owing to white cloak phenomena, muddying response in the clinic-setting [5]. From the trial management perspective, real-time monitoring may enable early identification of safety, operational, and compliance issues. Not surprisingly, applications for wearable technologies have already been demonstrated in a wide spectrum of disorders, including cardiovascular, respiratory, metabolic, psychiatric, and neurological disease [6]. Eventually, these innovations have the potential to evolve into regulatory-approved, clinical trial endpoints [7, 8].

Parkinson’s disease (PD) and Huntington disease (HD) are both chronic, neurologically-based movement disorders. Motorically, PD is characterized by a diverse set of symptoms that present at successive stages of the disease, including slowing of gait, shuffling feet, reduced arm swing, freeze of gait, asymmetry, tremor, bradykinesia, and dyskinesia. In HD, the most notable movement impairment is chorea, which is often similar to PD dyskinesia but is generally stable in contrast to PD symptoms that may fluctuate throughout the day. Given their chronic nature, lack of adequate therapies, and unique motor aspects, PD and to a lesser extent HD have been the focus of many wearable studies in disease [7, 9].

Remote trials with smartphones and wearable devices have been conducted in movement disorders such as Parkinson’s disease (PD) and Huntington disease (HD). Beyond a large collection of mostly short-duration studies aimed at quantifying symptoms [10], several efforts have examined longer-term, home-based monitoring in PD [11–16]. Four notable examples of large, multi-month, PD efforts that assessed aspects of remote compliance are the Parkinson@Home study which quantified the daily duration in which a patient-worn smartwatch was streaming data over a period of up to 13 weeks [13, 17], the mPower and HopkinsPD studies which published results on smartphone app usage and performance of home-based tasks over six months [14, 15], and the SMART-PD study which evaluated the impact of a smartphone app on medication adherence over four months [16]. In contrast, in HD existing remote studies have primarily focused on feasibility or symptom quantification over several days [9, 18, 19], as HD is an orphan disease which is naturally more challenging to recruit.

Despite its inherent advantages, remote patient-based data collection is prone to unique adoption and compliance issues [6, 20]. Technical factors include the need for frequent device charging, ease of device operation, and the simplicity of the user interface. Burden factors unique to remote study protocols include, for example, the imposition of recurrent performance of home-based tasks or requirement for consistent reporting on symptoms and drug intake times. These issues are notable, as often a high number of patients dropout of studies prior to completion, even in ‘traditional’ trials [3]. Moreover, the effect of multi-month duration is important as patient compliance in clinical trials of chronic conditions is lower than in acute conditions [21]. Collectively, this highlights the importance of understanding compliance dynamics and patient preferences with respect to the requirements of remote digital trials. Motivated by above, we analyzed two six-month studies of neurological movement disorders to better understand patient compliance patterns in remote settings for four digital study protocol metrics.

## Methods

**Fig. 1:**
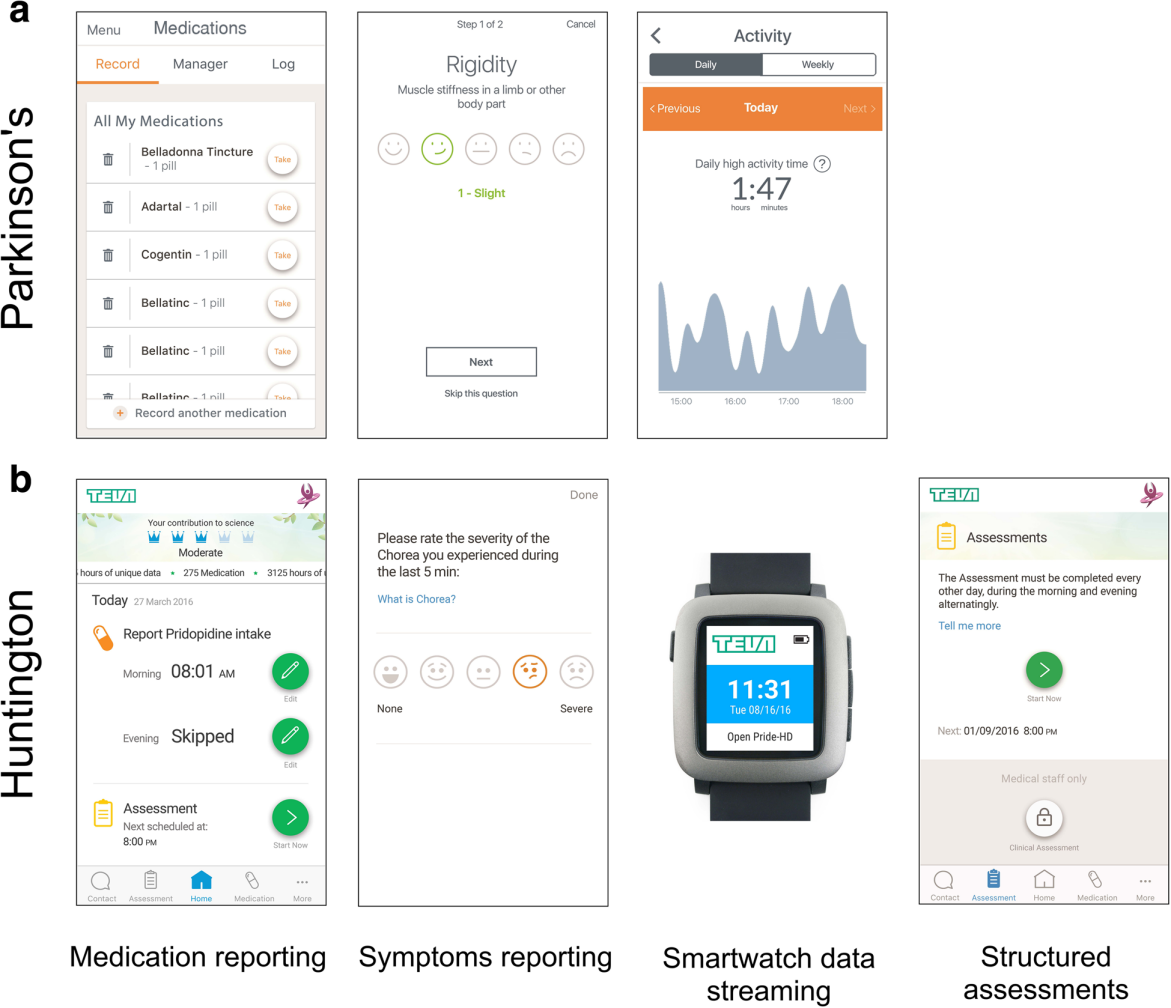
The four remote protocol compliance metrics tracked in this report using the Intel® Pharma Analytics Platform with their associated smartphone application screenshots. **a** The PD trial tracked three metrics as defined by the study protocol: app-based medication reporting of the patient’s normal, predefined medication regimen, smartwatch data streaming excluding charging time, and app-based daily symptoms reporting. **b** The HD trial tracked four compliance measures as defined by the study protocol: app-based medication reporting of the investigational drug, daily smartwatch data streaming excluding charging time, app-based daily symptoms reporting, and performance of structured motor assessments at home. The smartwatch graphic was obtained from Wikimedia Commons

Two independent clinical trials were analyzed: the Clinician Input Study in PD patients (CIS-PD) and the observational digital health sub-study within Study TV7820-CNS-20016 in HD patients (Open PRIDE-HD) (Table 1 and Table 2, respectively). The CIS-PD data used in the preparation of this article were obtained from the Michael J. Fox Foundation database. The total duration of each study was six months, and both studies used the Intel® Pharma Analytics Platform for data collection, monitoring, and analysis [17, 22]. The mobile application interface was designed using User Interface/User Experience (UI/UX) expert input to emphasize ease of use (Fig. 1).

**Table 1:** Characteristics of the PD and HD studies

Parameter	CIS-PD study	Open PRIDE-HD sub-study
Patient sample size	51	17
Study duration	6 months	6 months
Remote compliance metrics	(1) Medication reporting (2) Symptoms reporting (3) Smartwatch data streaming	(1) Medication reporting (2) Symptoms reporting (3) Smartwatch data streaming (4) Structured motor assessments at home
Sites	4 sites in US	11 sites across US, UK, Austria, Germany
Devices	Apple Watch, iPhone	Pebble watch, iPhone
App-based medication reporting	The normal medication regimen of the patient	Pridopidine (interventional investigational drug), twice per day per study protocol: a morning dose between 7:00 and 12:00, and an evening dose 7 to 10 h later
App-based symptoms reporting	Symptom severity three times per day	Chorea severity during the last five minutes, once per day
Wearing of smartwatch	A minimum of 12 h per day for 25 days per month	Continuously throughout study duration, preferably between 9:00–21:00
Performance of structured motor assessments at home	NA	Every other day, alternating mornings and evenings

**Table 2:** Demographics and disease status of the PD and HD patients. MDS-UPDR: Movement Disorder Society-Unified Parkinson’s Disease Rating Scale. UHDRS-TMS: Unified Huntington Disease Rating Scale Total Motor Score

Demographics	CIS-PD study	Open PRIDE-HD sub-study
Total patients	51	17
Gender: males	29 (57%)	9 (53%)
Ethnicity: Caucasian	45 (88%)	16 (94%)
Ethnicity: Hispanic	1 (2%)	1 (16%)
Age (mean ± s.d.)	62 ± 11	51 ± 12
Years since symptom onset (mean ± s.d.)	9 ± 5	NA
Years since diagnosis onset (mean ± s.d.)	7 ± 5	NA
PD: Hoehn & Yahr (mean ± s.d.)	2 ± 0.42	NA
PD: MDS-UPDRS Part 1 at baseline (mean ± s.d.)	10 ± 5	NA
PD: MDS-UPDRS Part 2 at baseline (mean ± s.d.)	10 ± 5	NA
PD: MDS-UPDRS Part 3 at baseline (mean ± s.d.)	24 ± 11	NA
HD: UHDRS-TMS	NA	37 ± 14
HD: Number of CAG repeats	NA	44 ± 3
HD: Neuroleptic use	NA	2 (12%)
HD: UHDRS-Total Functional Capacity	NA	8 (2)

### Parkinson’s disease trial

The CIS-PD trial was an observational study conducted in 4 US sites: Northwestern University, the University of Cincinnati, the University of Rochester, and the University of Alabama [23]. The study recruited 51 participants between June 2017 and August 2017, with 36 patients completing the entire study. Eligibility criteria included diagnosis of idiopathic PD, age 18 years old or older, Hoehn & Yahr stage 1–3, informed consent, and lack of cognitive impairment that would preclude study participation. Study participants used their own iPhone smartphone and were provided an Apple Watch smartwatch containing the Intel® Pharma Analytics Platform application. In-clinic visits occurred at baseline, after 2 weeks, after 1 month, after 3 months, and 6 months after baseline, during which clinicians performed assessments and reviewed a dashboard that dynamically displayed data from the smartwatch and smartphone application. The study also had a remote component as described below.

We tracked the three protocol-defined metrics related to remote participation: (1) app-based medication reporting, (2) smartwatch data streaming, and (3) app-based daily symptoms reporting (Fig. 1). We define smartwatch data streaming as the hours in which the smartwatch was broadcasting accelerometer data, not including charging time. Ideally most of this time reflects the hours in which the watch was worn by the patients, although if the watch was turned on, was not charging, and was not being worn those hours would be counted as well. Patients were instructed to follow their normal medication regimen and report on all medication intakes via the dedicated smartphone application throughout the duration of the study. Patients were asked to enter their medication schedule, which was used to prompt daily medication intake reminders on the smartphone. In addition, patients were instructed to wear the smartwatch for a minimum of 12 h per day for a minimum of 25 days per month. Finally, patients were asked to report their symptom severity three times per day in the dedicated smartphone application. The eight possible symptoms consisted of tremor, dyskinesia, rigidity, bradykinesia, gait problems, balance problems, voice problems, and constipation.

The PD trial incorporated planned support intervention calls and unplanned calls in the case of low compliance. Planned calls were scheduled at time points between the 1, 4, and 5-month in-clinic visits (±7 days) to reinforce compliance. Unscheduled telephone calls were made if patients were less compliant than expected with regards to smartwatch data streaming. In addition, the mobile phone application enabled patients to contact technical support via email or phone if needed.

### Huntington disease trial

The HD trial was an observational sub-study within the larger Open PRIDE-HD open-label, phase 2 trial (ClinicalTrials.gov NCT02494778), and was conducted in 4 countries: the United States, the United Kingdom, Austria, and Germany. The primary eligibility criteria were participation in the larger Open PRIDE-HD study, informed consent, willingness to comply with study requirements, and demonstrated capability to use the smartwatch device and smartphone application. The study recruited 17 patients (instead of the planned total of 60 patients due to early termination of the study) between December 2016 and December 2017, with 9 patients completing the entire study. Study participants were provided an iPhone 6 Plus smartphone and a Pebble smartwatch containing the Intel® Pharma Analytics Platform application. Besides two in-clinic visits, the study included a remote component as described below.

Within the trial, we tracked the four protocol-defined metrics related to remote participation: (1) app-based medication reporting, (2) smartwatch data streaming, (3) performance of structured motor assessments at home, and (4) app-based daily symptoms reporting (Fig. 1). In contrast to the PD trial, the HD trial had a home assessments component which did not exist in the former trial. First, patients were asked to take the investigational drug, pridopidine, twice per day per the main study protocol and report intake times using the dedicated smartphone application. Specific instructions were to take the morning dose between 7:00 and 12:00 and the second dose 7 to 10 h afterwards. Second, patients were instructed to continuously wear the smartwatch on the wrist of the chorea dominant upper-limb, preferably between 9:00 and 21:00. Third, participants were asked to perform a short, structured motor assessment at home every other day, alternating mornings and evenings, comprising two pre-defined tasks: standing still for 30 s and sitting at rest for 2 min with arms relaxed. Patients received reminders at the time of assessment (default settings at 9:00 and 18:00, alternating bi-daily) and were able to edit the reminder times. Fourth, patients were asked to report their chorea severity during the last five minutes once a day at a pre-defined time (default reminder at 12:00, time could be edited) using the dedicated smartphone application.

A phone call intervention mechanism was used to improve compliance rates. Support personnel performed monitoring of patient compliance twice a week to ensure fulfillment of two criteria: (1) performance of three structured motor assessments at home over the previous eight days and (2) having a minimal smartwatch data streaming score of three points over the previous three days. The score was calculated by defining a compliant day as having smartwatch data streaming for at least 90% of the waking hours (defined as 9:00 to 21:00), and then summing the score of the last three compliant days using the following values for each day: 1 point (for three days ago), 2 points (two days ago), and 4 points (one day ago). Non-compliant days were scored as 0. Support personal contacted the relevant site for patients that did not fulfill one of the above criteria, which in turn contacted the patient by phone within a few days. In addition, the mobile phone application enabled patients to contact technical support via email or phone if needed.

### Compliance metrics calculations

All metrics were calculated per day and presented smoothed using a moving average with a seven-day sliding window. The duration of smartwatch data streaming was defined by hours of streaming accelerometer data per day, excluding times in which the smartwatch was being charged. To be considered, each streaming hour required at least 90% of the expected number of records according to the accelerometer sampling rate, which was 50 Hz in both studies. Medication reporting rates considered both “take” or “skip” as a report. In the PD study, each symptom reporting event could contain between one and eight different symptoms as described above but was considered a single event regardless since it reflected a single interaction with the app.

## Results

### Longitudinal patterns

**Fig. 2:**
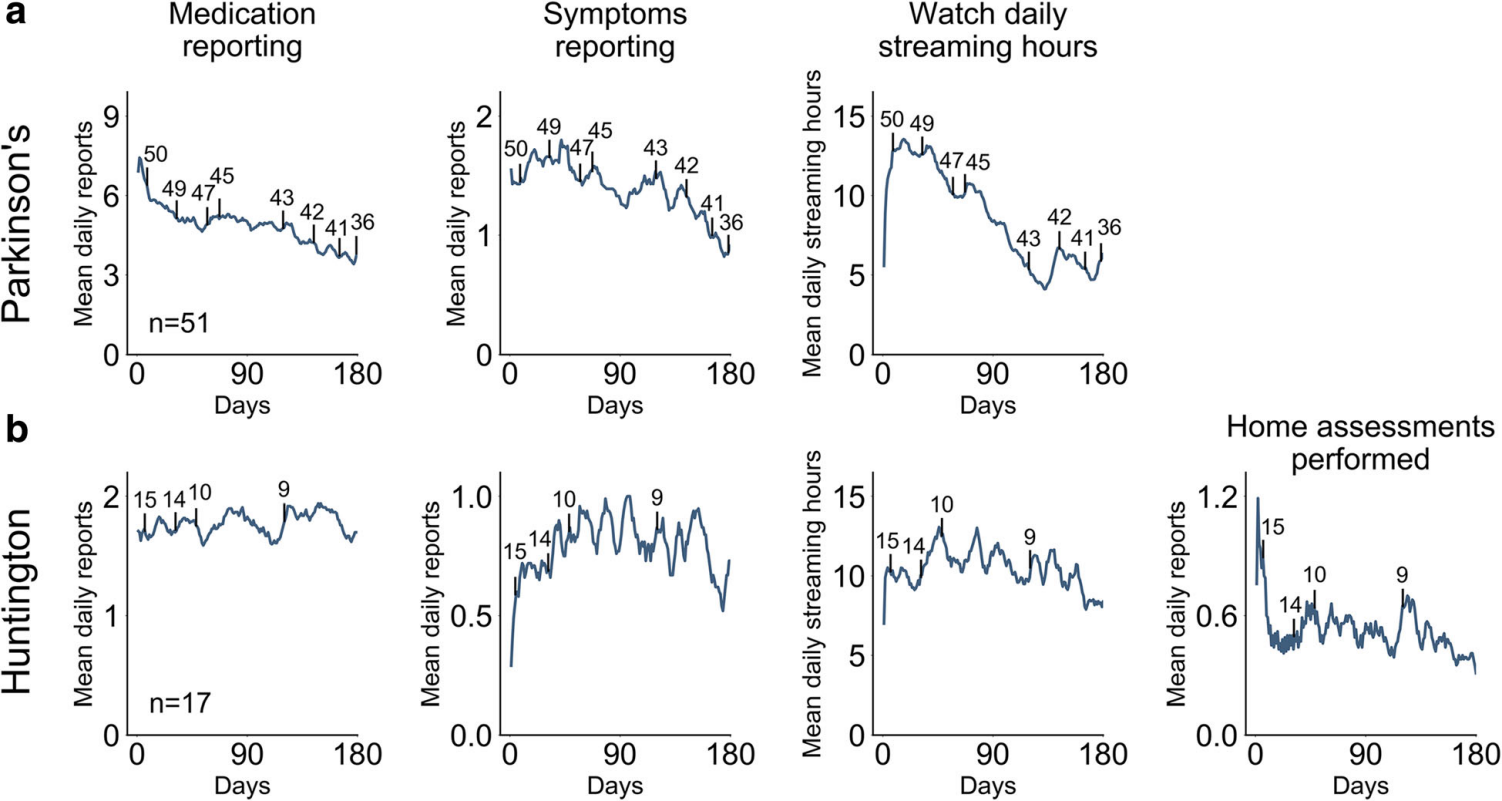
Aggregated compliance rates throughout studies. Mean longitudinal compliance levels for the (**a**) PD and (**b**) HD studies are presented. For the PD study, the three remote protocol metrics that were evaluated are shown: daily app-based medication reporting, daily app-based symptoms reporting, and daily smartwatch data streaming. The HD study also included bi-daily performance of structured motor assessments at home beyond the three former metrics. Vertical black lines represent censored data (patients that dropped out of the study), with the number above indicating the amount of patients remaining in the study. Vertical lines are plotted at select intervals to facilitate plot readability

We first looked at the aggregated patient compliance rates throughout both studies (Fig. 2). Three compliance metrics were tracked in the PD study and four in the HD study as the latter had an a structured motor assessments at home component which did not exist PD study. We quantify compliance rates as the extent to which the remote study protocol requirements were fulfilled. We observed that compliance rates were markedly distinct during the first few days as compared to the remaining duration of the studies. The duration of smartwatch data streaming was low during the first days for both studies. Initial app-based daily symptoms reporting rates were low and structured motor assessments at home compliance rates were high in the HD study. Given the early study fluctuations, we quantified longitudinal patterns starting at day 14.

Compliance rates gradually declined in the PD study. Mean daily app-based medication reporting rates dropped 34.2% from 5.82 to 3.83 (mean during study of 4.85). Mean daily symptom reporting events dropped from 1.61 events to 0.91, a 43.5% decline, with the mean of 1.39 being roughly half of the protocol requirement of 3 daily reports. Finally, the daily smartwatch data streaming hours declined 52.7% from 13.32 h to 6.3 h (mean during study of 8.6 h).

The compliance rates in the HD study varied in their dynamics. Both daily app-based medication reporting and symptoms reporting remained relatively constant. For medication reporting, values decreased only from 1.75 to 1.7 daily reports (2.86% drop). This is close to the expected 2 daily reports. Mean daily symptoms reporting slightly increased by 1.39% from 0.72 daily reports on day 14 to 0.73 on the final day of the study (study mean of 0.79 daily reports). In contrast, smartwatch data streaming hours decreased 20.36% from 10.51 h to 8.37 h (mean of 10.45 h for entire study). Finally, the amount of structured motor assessments at home performed declined 30.37% from 0.95 every two days to 0.66 every two days (mean during study of 1.04 every two days) (Fig. 2 shows the daily rather than bi-daily values for plotting consistency). In both studies, compliance rates were similar among patients who completed the study and those who terminated early (Additional file 1 Figure S1).

### Compliance variation per patient

**Fig. 3:**
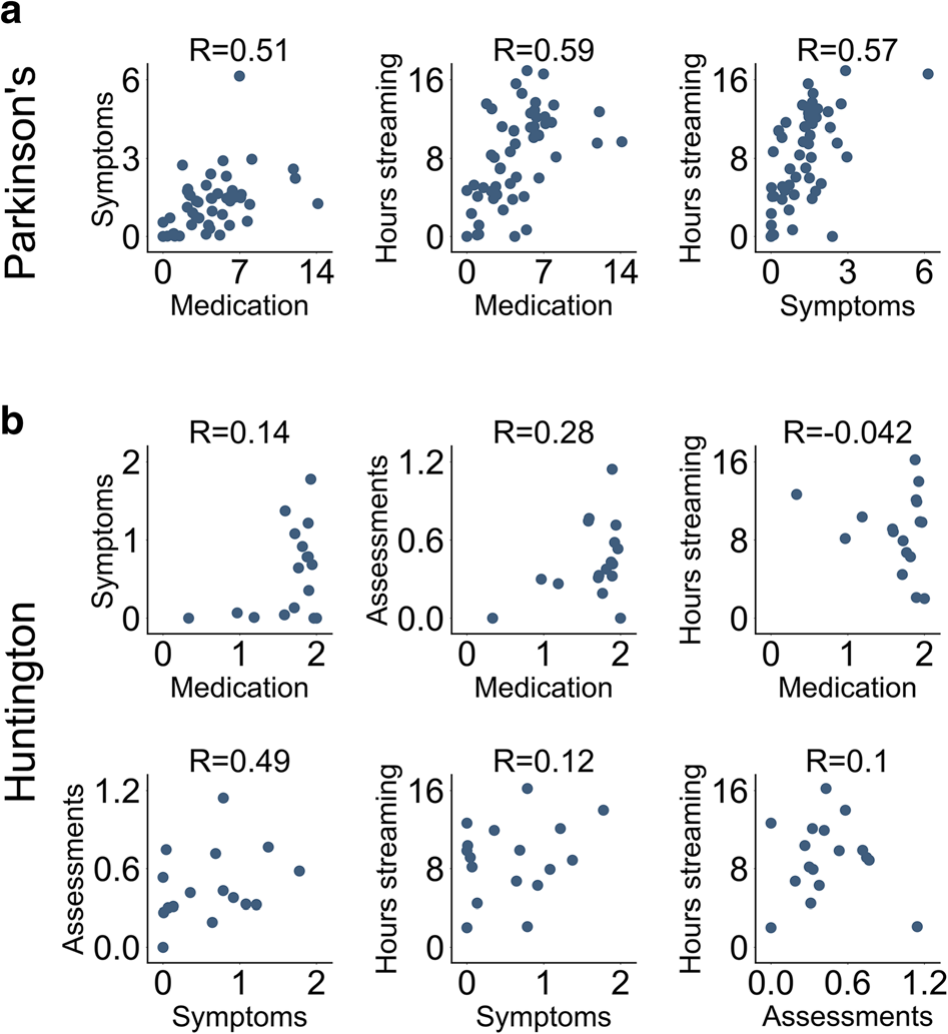
Individual variation in remote study protocol compliance metrics. Scatter plots depict the pairwise association for all compliance metric pairs. Each circle represents an individual patient, specifically the mean compliance rate for that patient. Data shown for the (**a**) PD and (**b**) HD studies. The distribution for individual metrics can be observed by looking at each individual axis. The R values in the plot are Spearman’s rank-order correlations. The positive correlations suggest that individual patients tend to have either high or low compliance rates across multiple remote study compliance metrics. Axis labels: Symptoms (mean daily symptom reporting events per patient), Medication (mean daily medication reporting events per patient), Hours streaming (mean smartwatch daily streaming hours per patient), and Assessments (mean daily structured motor assessments at home reported by patient). Axis values are counts or hours where appropriate

An examination of individual compliance rates showed that patients generally had either high or low compliance across all metrics (Fig. 3). This is evident, especially in the PD study, as the compliance metric pairs had similar degrees of positive correlations in individual compliance rate (PD metric pairwise correlations: 0.51, 0.57, and 0.59). The effect is stronger in the PD study, perhaps due to higher patient sample size.

### Daily and hourly preferences

**Fig. 4:**
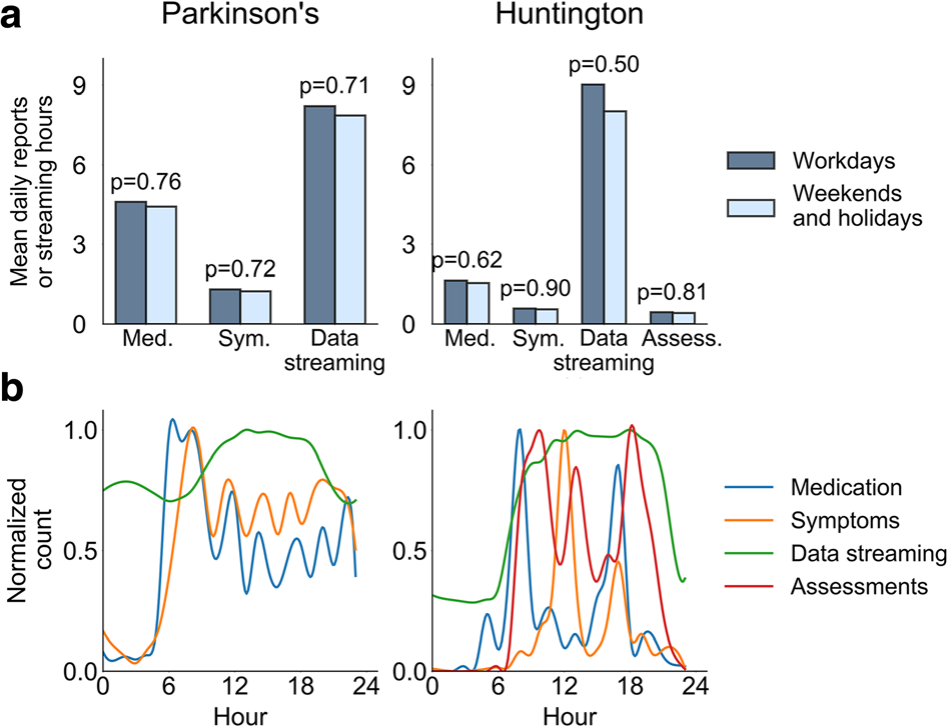
Hourly and daily compliance patterns. (**a**) Hourly and (**b**) daily compliance levels are portrayed for all three remote compliance metrics studied in the PD study and the four metrics studied in the HD study. The hourly patterns may reflect personal preferences and/or the impact of predefined schedules, as determined by the study protocol, or predefined reminders. Med. – app-based medication reporting, Sym. - app-based symptoms reporting, Data streaming - smartwatch data streaming, Assess. - performance of structured motor assessments at home

We next studied the impact of daily and hourly preferences with respect to the remote protocols. We found that the impact of weekends and holidays on compliance rates was minimal, with only a slight, statistically nonsignificant, decrease in smartwatch data streaming observed in both trials (HD *p* = 0.50, PD *p* = 0.71, two-tailed Student’s T-test) (Fig. 4a). Likewise, there were no differences among gender in both studies (Additional file 2 Figure S2), subject age only had a minor correlation with most compliance metrics (Additional file 3 Figure S3), and baseline disease status had only a small correlation with most metrics as well (Additional file 4 Figure S4). The exception was daily smartwatch data streaming hours in the HD study which had positive correlation with age (*R* = 0.46) and baseline UHDRS-TMS rating (*R* = 0.49) (Additional file 4 Figure S4).

In contrast, there was substantial variation in hourly compliance rates. This was expected given the predefined schedules for medication intake in both studies and for app-based symptoms reporting and structured motor assessments at home in the HD study (Fig. 4b). Beyond predefined times, we observed that PD patients preferred reporting on symptoms during the morning hours. Finally, in both studies, smartwatch data streaming rates gradually increased during the morning and peaked at around noon. The level then remained relatively constant until around 20:00 in the HD study, whereas in the PD trial there was a decrease during the afternoon and early evening hours.

## Discussion

Remote monitoring is expected to gradually transform drug development procedures, with assessment of patient compliance using digital monitoring being increasingly useful for smart trial design. The current report includes a relatively long-duration follow-up of movement disorder patients from two unrelated trials with respect to four compliance measures that are specific to remote trials.

Several of our findings may assist in the design of similar clinical trials in the future. First, we observed that the variability of the compliance rates at the beginning of the trial are non-indicative of compliance patterns for the vast-majority of the remainder of the trial duration. This is not surprising given the need to adjust to new technology and may need to be considered during trial planning. Second, when given the option, study participants preferred to report their symptoms during the morning hours. Third, defining appropriate eligibility criteria and screening methods appears to be especially important in remote, technology-based trials given that compliant patients tend to exhibit high compliance across the different aspects of the remote trial protocols, rather than to only to a single protocol metric. This is likely influenced by factors such as age, technology savviness, and various additional factors which require further research.

As expected, there were differences in compliance rates over time between the two studies. While smartphone app-based interactions have been shown to increase compliance [16], the observation of gradually decreasing engagement in the PD study was not surprising as decreased compliance over time has been shown in numerous trials, for instance in a trial in which 50% of patients stopped taking anti-hypertensive drug within one year [24]. One potential reason for the observed difference between the trials may be that the HD trial was interventional, perhaps increasing the motivation of patients seeking experimental drug benefits. In addition, the HD trial implemented a higher level of interventional support calls which may have maintained higher compliance levels.

In this work we attempted to understand the adherence of HD and PD patients to remote clinical trial protocols over a multi-month duration. It is important to note that we defined compliance as patient participation levels rather than compliance as strictly defined by the study protocols. While both measures are tightly linked, participation levels are continuous and are thus better suited for understanding patient behavior. In contrast, protocol-defined compliance is often binary due to the definition of thresholds. For example, the PD study protocol defined smartwatch data streaming compliance as streaming data for at least 10 h per day for 25 days per month.

While HD is an orphan disease with little or perhaps no precedent of studies examining remote compliance, our work is not the first to investigate remote compliance in PD. In the large observational Parkinson@Home study, mean daily smartwatch data streaming durations of 14.8 h – 16.3 h were observed for two large cohort studies of 6 weeks (cohort 1, *n* = 304) and 13 weeks (cohort 2, *n* = 649) [13]. As in our PD trial, the researchers observed only a mild reduction in smartwatch data streaming of roughly 25% from start to finish, which they proposed may be attributed to the passiveness of, or the lack of interaction needed for, data collection. Another study, titled mPower, focused on data collection from thousands of users using home-assessments that leverage smartphone sensors and a corresponding mobile app [14]. In a paper describing the first six months of data collection, the authors observed a rapid, exponential-like drop in average data contribution per patient, which could be due to many factors including the remote recruitment, no interventional treatment, and no support or trial management intervention. Another effort, HopkinsPD collected passive data from the smartphones of PD patients and control subjects and asked for performance of two structured motor assessments at home during the morning hours [15]. While structured assessment compliance relative to protocol was not reported, the authors showed that the abundance of structured assessments at home as well as passive usage of the smartphone remained uniform across all days of the week, similar to our findings.

Patient compliance is a broad topic with many aspects and research challenges. One major challenge is obtaining large patient samples sizes given costs and recruitment challenges, especially in rare diseases. Our HD study had 17 patients, whereas a larger number may potentially impact conclusions. Despite the small sample size, this study provides an example for monitoring patient compliance in long-duration remote studies and can be used in the design, planning and execution of future studies in this patient population. Additionally, factors not addressed in this work such as mobile application user experience and interface design may influence compliance rates. Finally, a deeper analysis of digital trial economics, including device cost, data management and clinic visit savings, can help illustrate the financial considerations in such trials.

## Conclusions

Understanding patterns of patient compliance in remote, technology-based clinical trials requires analysis of data collected using digital technologies. The insights from our work suggest that such remote trials are feasible, even when comprising multiple protocol requirements. Beyond our observations, examination of additional factors that impact compliance such as the impact of support, intervention, alternative application design interfaces, and additional covariates can further influence study design.

In the broader perspective, this report supports the growing trend of using mobile applications and wearable technologies to monitor, prompt, and encourage patient compliance with medication intake and performance of clinical assessments. This effort strengthens the notion that these data have the potential to provide further insight regarding patients’ daily lives outside the clinic and potentially evolve into novel endpoints for regulatory purposes.

## Additional files

Additional file 1: **Figure S1.** Comparison of compliance rates throughout studies between early dropouts and patients that completed the study (DOCX 150 kb)

Additional file 2: **Figure S2.** Compliance patterns by gender for the PD and HD studies (DOCX 87 kb)

Additional file 3: **Figure S3.** Compliance patterns by age for the PD and HD studies (DOCX 109 kb)

Additional file 4: **Figure S4.** Compliance patterns by baseline disease status for the PD and HD studies (DOCX 103 kb)

## Abbreviations

CAG: Cytosine Adenine Guanine
CIS-PD: Clinician Input Study in PD patients
HD: Huntington disease
hrs: Hours
Hz: Hertz
IRB: Institutional Review Board
MDS-UPDRS: Movement Disorder Society-Unified Parkinson’s Disease Rating Scale
NA: Not available
Open PRIDE-HD: The observational digital health sub-study within Study TV7820-CNS-20016 in HD patients that evaluated pridopidine
PD: Parkinson’s disease
s.d: Standard deviation
UHDRS-TMS: Unified Huntington Disease Rating Scale Total Motor Score
UI/UX: User Interface/User Experience
