# Supplementary material for: Characterizing patient compliance over six months in remote digital trials of Parkinson’s and Huntington disease
Source: BMC Med Inform Decis Mak. 2018 Dec 20;18:138. doi: 10.1186/s12911-018-0714-7 (PMC6302308; doi:10.1186/s12911-018-0714-7)
Supplement: Supplementary file 1 — Figure S1. Comparison of compliance rates throughout studies between early dropouts and patients that completed the study (DOCX 150 kb) [file 12911_2018_714_MOESM1_ESM.docx]

**
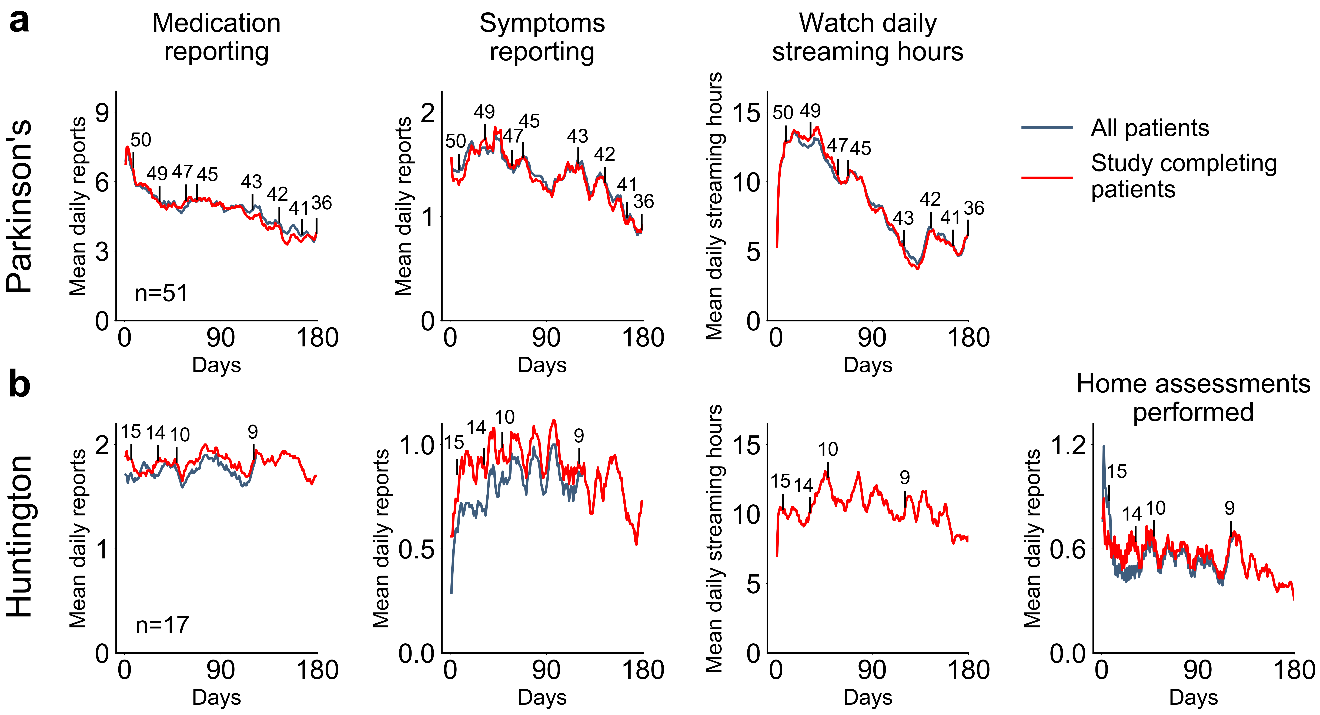
**

**Supplementary Fig. 1** Comparison of compliance rates throughout studies between early dropouts and patients that completed the study. Mean longitudinal compliance levels for the (**a**) PD and (**b**) HD studies are presented. Compliance levels are shown for all patients (black line) as well as only for those that completed the study (red line). Four metrics are shown: daily app-based medication reporting, daily app-based symptoms reporting, daily smartwatch data streaming, and bi-daily performance of home assessments (HD only). Only the former three metrics were evaluated in the PD study as home assessments were not part of the study protocol. Vertical black lines represent censored data (patients that dropped out of the study), with the number above indicating the amount of patients remaining in the study. Vertical lines are plotted at select intervals to enable plot readability.
