# Supplementary material for: Characterizing patient compliance over six months in remote digital trials of Parkinson’s and Huntington disease
Source: BMC Med Inform Decis Mak. 2018 Dec 20;18:138. doi: 10.1186/s12911-018-0714-7 (PMC6302308; doi:10.1186/s12911-018-0714-7)
Supplement: Supplementary file 2 — Figure S2. Compliance patterns by gender for the PD and HD studies (DOCX 87 kb) [file 12911_2018_714_MOESM2_ESM.docx]

**
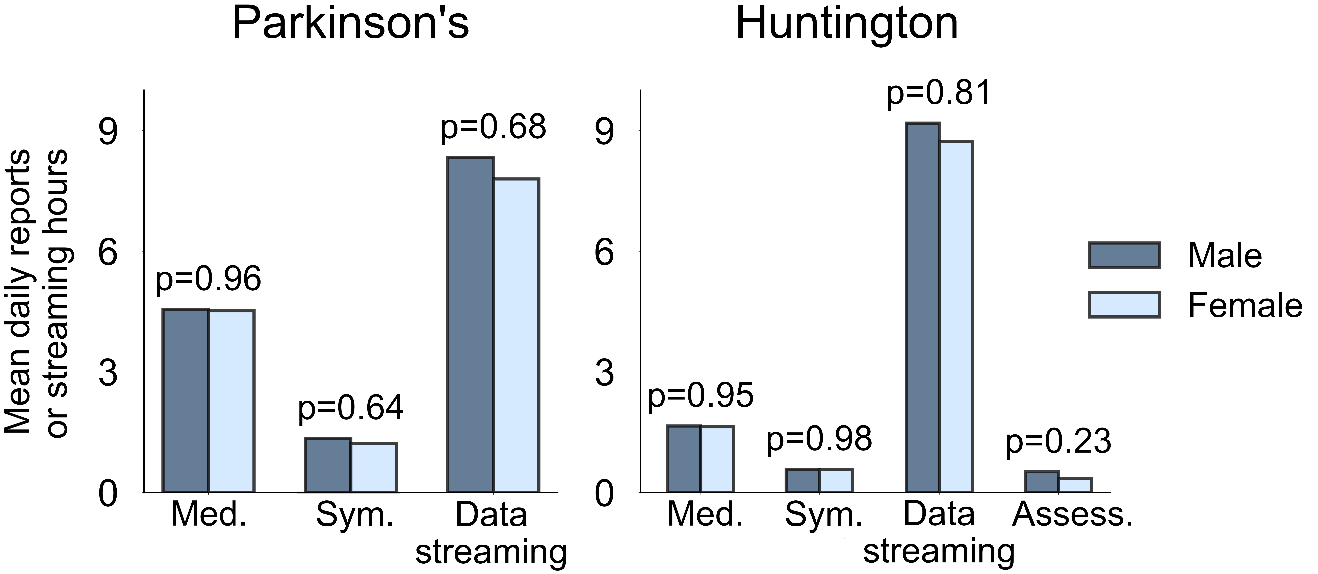
**

**Supplementary Fig. 2** Compliance patterns by gender for the PD and HD studies. Compliance patterns are portrayed for all three remote compliance metrics studied in the PD study and the four metrics studied in the HD study. For each metric, no gender differences were observed within each study. Med. – app-based medication reporting, Sym. - app-based symptoms reporting, Data streaming - smartwatch data streaming, Assess. - performance of structured home motor assessments
