# Supplementary material for: Characterizing patient compliance over six months in remote digital trials of Parkinson’s and Huntington disease
Source: BMC Med Inform Decis Mak. 2018 Dec 20;18:138. doi: 10.1186/s12911-018-0714-7 (PMC6302308; doi:10.1186/s12911-018-0714-7)
Supplement: Supplementary file 3 — Figure S3. Compliance patterns by age for the PD and HD studies (DOCX 109 kb) [file 12911_2018_714_MOESM3_ESM.docx]

**
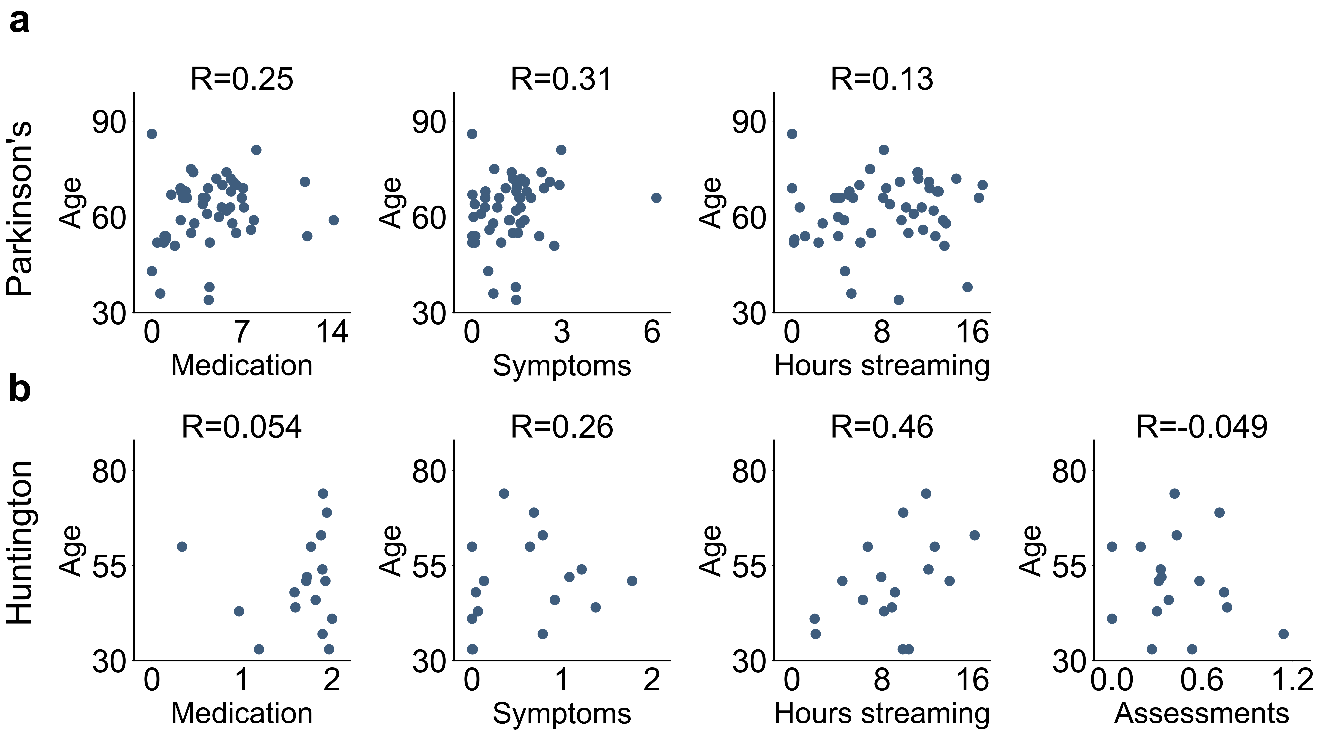
**

**Supplementary Fig. 3** Compliance patterns by age for the PD and HD studies. Scatter plots portray compliance patterns for all three remote compliance metrics studied in the PD study and the four metrics studied in the HD study. The R values in the plot are Spearman’s rank-order correlations.
